# Standardized Approach for Laparoscopic Hemispheric Liver Resection for Segments 7 and 8

**DOI:** 10.1002/deo2.70203

**Published:** 2025-09-02

**Authors:** Ryoichi Miyamoto, Masahiro Shiihara, Mitsuru Watanabe, Jiro Shimazaki, Mitsugi Shimoda, Shuji Suzuki

**Affiliations:** ^1^ Department of Gastroenterological Surgery, Ibaraki Medical Center Tokyo Medical University Ibaraki Japan

**Keywords:** anatomical resection, laparoscopic hemispheric liver resection, laparoscopic liver resection, LLR, segments 7 and 8

## Abstract

**Background:**

We investigated whether the standardized “can‐opener method” surgical technique is an adequate surgical procedure for hemispheric hepatectomy in segments 7 and 8.

**Methods:**

Forty‐two patients who underwent laparoscopic hemispheric liver resection for segments 7 and 8 using our standardized surgical technique were enrolled. To examine the effect of this standardized surgical procedure on short‐term outcomes, patients were classified into two groups based on the timing of the standardization of their procedures (Group A, the first half of the cases, and Group B, the second half of the cases). Short‐term outcomes were subsequently compared between the two groups.

**Results:**

Significant differences in operation time (465 min vs. 332 min, *p* = 0.001), intraoperative blood loss volume (645 g vs. 105 g, *p* = 0.011), postoperative complications (Clavien‒Dindo Grade I and Grade II) (7 vs. 1, *p* = 0.011), and length of postoperative hospital stay (10 days vs. 7 days, *p* = 0.001) were detected between the two groups. All patients had negative surgical margins. With respect to postoperative complications, four patients had Grade I complications, such as wound infection and minor pneumonia, and four patients had Grade II complications, such as bile duct infection and intra‐abdominal abscess. No patients experienced 90‐day mortality.

**Conclusion:**

Our standardized surgical technique is an adequate surgical procedure for hemispheric hepatectomy in segments 7 and 8 and is referred to as the “can‐opener method”.

## 1 Introduction

Although laparoscopic liver resection (LLR) was initially reported by Reich et al. in 1991, laparoscopic procedures were not widely accepted due to the difficulty in controlling bleeding [1, 2, 3]. Improvements in laparoscopic techniques and instruments, as well as accumulated experience, have led to their widespread acceptance within the surgical community as a precise and safe alternative to open procedures [4, 5, 6]. The international position on LLR was addressed by experts in hepatobiliary surgery at an international consensus conference in Louisville, Kentucky (USA), in November 2008 [4]. These experts concluded that LLR is considered a safe and effective approach when performed by experienced surgeons.

In the evolution of LLR, the lesions located in the upper part of the right anterior sector and in the posterior sectors, segments 7 and 8, were initially considered extremely difficult for laparoscopic surgery due to the limited visualization in relation to the diaphragm and ribs, the greater risk of bleeding and difficult control associated with a higher transfusion requirement, the higher conversion rate in the early series, the longer operative time, the greater difficulty in obtaining surgical margins and the greater technical complexity of liver mobilization. This location, together with the difficulty of access, makes the technical aspects more relevant [7, 8, 9].

Therefore, the surgical approach for segments 7 and 8 remains a greater challenge due to their location in the deepest region of the abdominal cavity, together with their relationship to the hepatic veins and the large number of interconnected vascular branches from the Glissonean pedicles. To facilitate the surgical approach to these locations, different technical modifications have been proposed [10, 11, 12, 13, 14, 15]. Okuda et al. reported the efficacy of the intrahepatic Glissonean pedicle approach to segment 7 from the dorsal side during laparoscopic anatomic hepatectomy of the cranial part of the right liver [11]. Ome et al. also reported the efficacy of laparoscopic anatomic liver resection of segment 8 using the intrahepatic Glissonean approach [12]. Furthermore, Ichida et al. reported the efficacy of using intercostal trocars for laparoscopic resection of subphrenic hepatic tumors [13].

However, there are few reports on a standardized approach for hemispheric hepatectomy in segments 7 and 8, where there are few landmark vessels and the depth of dissection is complicated. Therefore, we have originally used a standardized surgical technique, the “can‐opener method”, for hemispheric hepatectomy in segments 7 and 8.

We investigated whether this standardized surgical technique is an adequate surgical procedure for hemispheric hepatectomy in segments 7 and 8. Furthermore, herein, we address the perioperative outcomes of patients who underwent this surgical procedure at a single center.

## 2 Materials and Methods

### 2.1 Patients

We retrospectively evaluated 109 consecutive patients who underwent LLR at Ibaraki Medical Center, Tokyo Medical University, Japan. The ethics committee of our institute approved this study. First, we excluded 60 patients who underwent surgeries that involved more extensive standard LLR procedures, such as anatomical resection, or who underwent surgeries for other lesions, excluding those in segments 7 and 8. Second, we excluded 3 patients whose tumors were located in the dorsal region of segment 7, whose hemispheric resection was difficult in terms of the surgical field of view. In terms of the approach to the dorsal region of segment 7, a dorsal approach was reported to be useful [10, 11, 12, 13, 14, 15]. Therefore, this study examined only cases in which it was possible to perform hemispherical resection from the front. In the final cohort, 42 patients who underwent laparoscopic hemispheric liver resection for segments 7 and 8 were enrolled. In our institution, laparoscopic hemispheric liver resection is indicated when the diameter of the tumor is less than 5 cm and when a resected margin of separation from the main Glisson's vessels can be observed. To examine the effect of standardizing surgical procedures on short‐term outcomes, patients were classified into two groups based on the timing of the standardization of their procedures (Group A, the first half of the cases, and Group B, the second half of the cases). Short‐term outcomes were subsequently compared between the two groups.

### 2.2 Surgical Procedure “Can‐opener Method”

**FIGURE 1:**
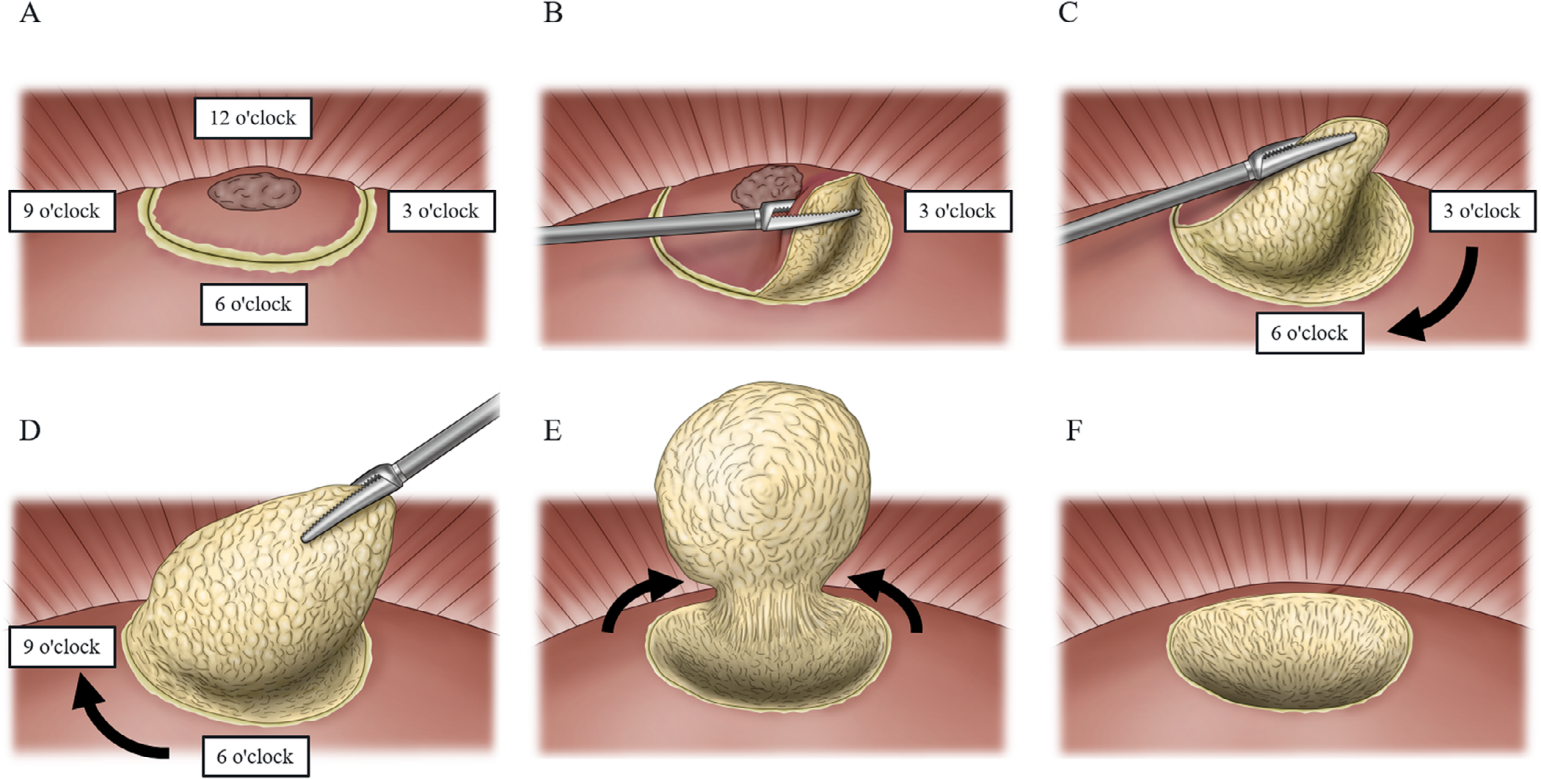
(A) This view is an anterior image from a liver tumor patient. We defined the direction of the hepatic dissection as clockwise when the hepatic dissection line was viewed from the front, with a margin from the tumor. The location of the liver resection surface was represented as a clock face, from 1 to 12 o'clock. (B) Liver dissection was initially performed at 3 o'clock. During resection at 3 o'clock, the liver was dissected with the goal of determining the adequate depth of dissection apart from the tumor. The depth of dissection was set at the 3 o'clock position as a lateral approach to tumors. (C) This image shows a liver dissection in a frontal view from 3 to 6 o'clock. Liver dissection proceeded with the goal of broadly connecting the dorsal margin by performing broad liver dissection from 3 to 6 o'clock on the anterior surface, targeting a predetermined dorsal dissection margin at 3 o'clock (black arrow). (D) This image shows a liver dissection in a frontal view from 6 to 9 o'clock. The assistant surgeon deployed the liver on the specimen side and performed hepatic dissection to connect the deep hepatic dissection margins from 6 to 9 o'clock (black arrow). (E) This image shows a liver dissection in a frontal view from both sides at 1 and 11 o'clock. The liver on the specimen side was elevated, and the sample was removed by hepatectomy from both sides at 1 and 11 o'clock (black arrows). This series of dissecting procedures resembles the action of opening a can, which is the reason why this surgical procedure was named the “can‐opener method”. (F) This image shows the liver dissection plane after hepatectomy. By leaving the liver capsule in place until the end of resection, it was possible to prevent the liver from being torn off.

We describe our surgical procedure, the “can‐opener method” for hemispheric hepatectomy (Figure 1). The concept of this surgical technique is to resect the liver clockwise, like opening a can. We define the direction of the hepatic dissection as clockwise when the hepatic dissection line is viewed from the front, with a margin from the tumor. The location of the liver resection surface was represented as a clock face, from 1 to 12 o'clock (Figure 1A). The term “can‐opener method” is not widely known. Rather, it is used exclusively at our facility. We use this terminology to help standardize surgical procedures.

**FIGURE 2:**
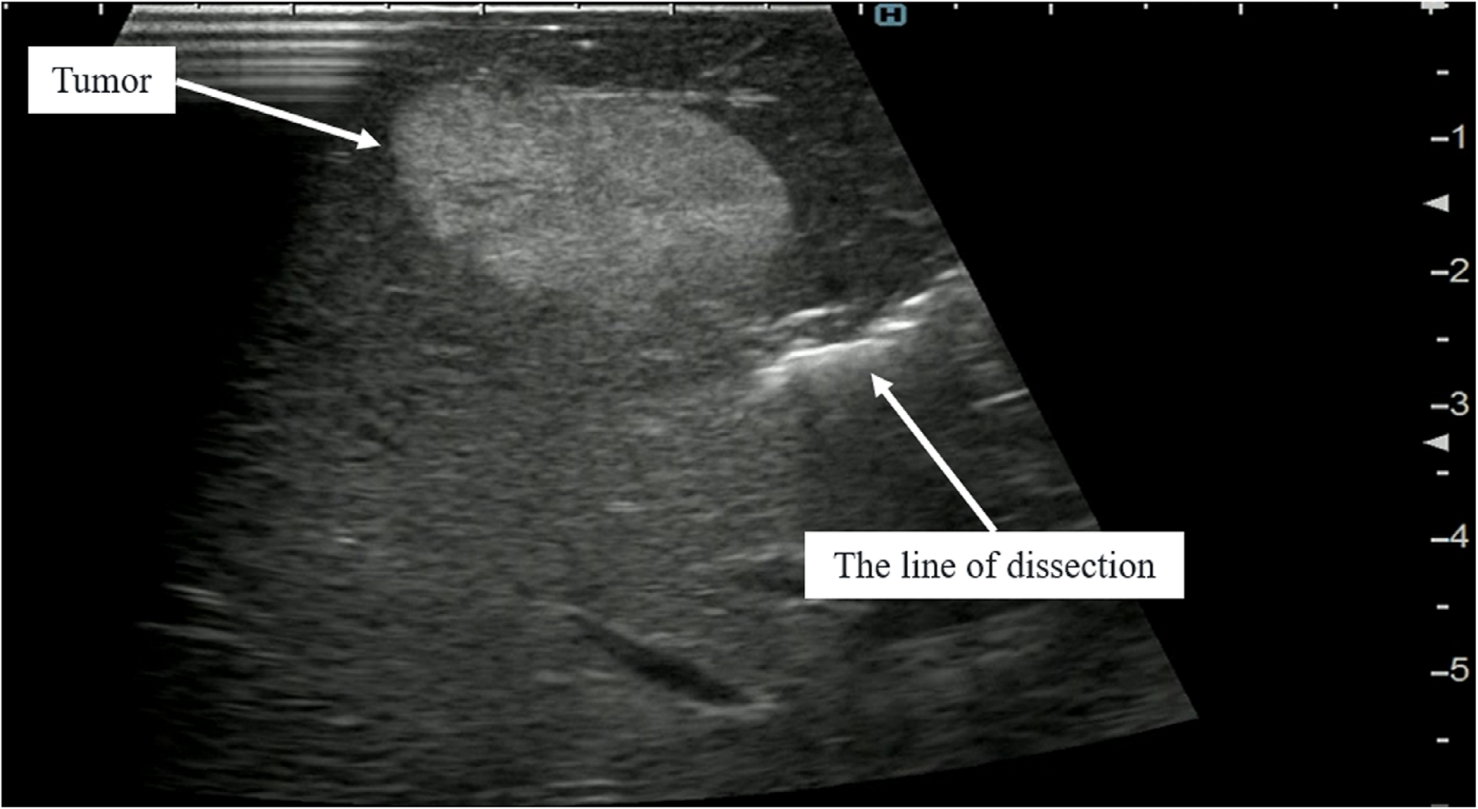
An image of intraoperative ultrasound during liver dissection is shown. Intraoperative ultrasound was performed as needed to assess the relationship between the tumor and the line of dissection and to confirm whether the deepest tumor margin had been secured.

First, we start the hepatectomy at 3 o'clock. During resection, the liver is dissected with the goal of determining the adequate depth of dissection apart from the tumor (Figure 1B). Intraoperative ultrasound is performed as needed to assess the relationship between the tumor and the line of dissection (Figure 2). When the resection line is being established, Glisson's trunk or the hepatic vein can serve as landmarks, if visible. When we recognize Glisson's trunk along the resection line, we also evaluate the ischemic area and determine whether it should be removed. Second, liver dissection proceeds with the goal of broadly connecting the dorsal margin by performing broad liver dissection from 3 to 6 o'clock on the anterior surface, targeting a predetermined dorsal dissection margin at 3 o'clock (Figure 1C). Third, liver dissection is performed along the deep dorsal dissection margin. The assistant surgeon deploys the liver on the specimen side and performs hepatic dissection to connect the deep hepatic dissection margins from 6 to 9 o'clock (Figure 1D). Finally, the liver on the specimen side is elevated, and the specimen is removed by hepatectomy from both sides at 1 and 11 o'clock. By leaving the liver capsule in place until the end of resection, it is possible to prevent the liver from being torn off (Figure 1E,F).

### 2.3 Short‐term Outcomes

For short‐term outcomes, we collected data concerning the operating time, intraoperative blood loss volume, intraoperative blood transfusion, conversion to laparotomy, and postoperative complications. Postoperative complications were also graded according to the Clavien–Dindo classification [16]. Furthermore, we focused on 90‐day mortality and the length of postoperative hospital stay. In terms of oncological outcomes, surgical margins were also analyzed.

### 2.4 Surgical Management

We routinely performed the laparoscopic technique with five ports, a 10‐mmHg pneumoperitoneum, and a flexible‐angle laparoscope in the left half lateral position. Intercostal trocar or hand‐assisted techniques were not routinely employed. In all the patients, the right adrenal gland was detached, and the right margin of the inferior vena cava (IVC) was completely exposed for right hepatic lobe mobilization. Pringle's maneuver was routinely used [17]. Parenchymal liver transection was performed with a THUNDERBEAT system (TS) (Olympus Medical Systems Corp., Tokyo, Japan) and a Cavitron ultrasonic surgical aspirator (CUSA: Integra Lifesciences Corporation, NJ, USA). Bipolar coagulation was used to treat minor bleeding. The resected liver was placed in a plastic bag and extracted, without fragmentation, through a small abdominal incision. A drainage tube was routinely placed on the resected liver surface. The methods of managing the drain and checking the total bilirubin levels in the drainage fluid were standardized in this study. The drain was removed if the drainage fluid was clear and if both bile leakage and bacterial contamination were absent. All patients received prophylactic antibiotics either intraoperatively or for 1 or 2 days postoperatively.

## 3 Results

### 3.1 Patient Characteristics

Patient characteristics between the two groups are shown in Table 1. In the present cohort, there were no significant differences in terms of age or sex ratio between the two groups. To assess liver function, the Child‒Pugh classification and the degree of liver damage, including the indocyanine green retention rate at 15 min, were employed. There were no significant differences in terms of liver function, including the Child‒Pugh classification or degree of liver damage, between the two groups. In terms of tumor size, tumor characteristics, number of lesions, and tumor location, there were no significant differences between the two groups. No patients with dorsal lesions of segment 7 were included in this study.

**TABLE 1:** Patient characteristics between the two groups.

Factors	Group A (*n* = 21)	Group B (*n* = 21)	*p*‐Value
Age	71 (34–83)	72 (41–84)	0.211
Sex ratio (male/female)	17/4	15/6	0.114
Child‐Pugh (A/B/C)	21/0/0	21/0/0	0.310
Liver damage (A/B/C)	19/2/0	20/1/0	0.230
Tumor size (mm)	27.3 ± 10.3	31.4 ± 13.5	0.661
Tumor characteristics			
HCC	15 (71%)	13 (62%)	
CRLM	5 (24%)	7 (33%)	0.314
ICC	1 (5%)	1 (5%)	
Number of lesions			
Single	16 (76%)	14 (67%)	0.448
Multiple	5 (24%)	7 (33%)	
Tumor location			
S7 (S7/8)	6 (29%)	8 (38%)	
S8	6 (29%)	5 (24%)	
S4/8	4 (19%)	2 (9.0%)	0.235
S5/8	3 (14%)	3 (14%)	
S4/5/8	2 (9.0%)	3 (14%)	

Abbreviations: CRLM, Colorectal liver metastasis; HCC, hepatocellular carcinoma; ICC, intrahepatic cholangiocarcinoma; S, Segment.

### 3.2 Short‐term Outcomes

The short‐term outcomes of the two groups are shown in Table 2. All patients who met our eligibility criteria underwent standardized laparoscopic hemispheric hepatectomy. All patients had negative surgical margins. Intercostal ports were used in only 2 patients whose tumors were large and difficult to expose. Significant differences were observed in terms of operating time (465 min vs. 332 min, *p* = 0.001), intraoperative blood loss volume (645 g vs. 105 g, *p* = 0.011), postoperative complications (Clavien‒Dindo Grade I and Grade II) (7 vs. 1, *p* = 0.011), and length of postoperative hospital stay (10 days vs. 7 days, *p* = 0.001).

**TABLE 2:** Short‐term outcomes between the two groups.

Factors	Group A (*n* = 21)	Group B (*n* = 21)	*p*‐Value
Operating time (minutes)	465 (225–655)	332 (175–338)	0.001^*^
Intraoperative blood loss volume (grams)	645 (455–1480)	105 (5–680)	0.011^*^
Intraoperative blood transfusion			
Yes/No	2/19	1/20	0.230
Conversion to laparotomy	1 (4.8%)	1 (4.8%)	1.000
Surgical margin			
Negative/Positive	21/0	21/0	1.000
Postoperative complications(C‐D grade)			
None/Grade I/II	14/3/4	20/1/0	0.025^*^
Grade III/IV	0/0	0/0	
90‐day mortality	0	0	1.000
Length of postoperative hospital stay (days)	10 (7–35)	7 (5–14)	0.001^*^

Abbreviation: C‐D, Clavien‐Dindo classification.

* *p* < 0.05.

With respect to postoperative complications, four patients had Grade I complications, such as wound infection and minor pneumonia, and 4 patients had Grade II complications, such as bile duct infection and intra‐abdominal abscess. No patients experienced 90‐day mortality.

## 4 Discussion

In this study, the short‐term outcomes obtained using our own standardized technique, named the “can‐opener method”, were satisfactory. Therefore, we found that our standardized surgical technique is an adequate surgical procedure for laparoscopic hemispheric hepatectomy in segments 7 and 8.

In terms of short‐term outcomes, two patients underwent conversion to laparotomy. One patient was being treated with anticancer drugs for colorectal liver metastases and was converted to laparotomy to control bleeding from the liver because the drug‐induced liver damage was more advanced than preoperatively expected. In the other case, the patient was converted to laparotomy because the intra‐abdominal adhesions from the previous surgery were so strong that it was difficult to perform Pringle's maneuver. In terms of postoperative complications, compared with those in previous reports, the frequency of postoperative complications and the type of complication are acceptable [18, 19].

Although many reports have been published on anatomical liver resection, formalizing the technique of hemispheric hepatectomy is difficult due to the difficulty in establishing landmark vessels or setting the depth and direction of the line of dissection; thus, few reports have been published on the method of laparoscopic hemispheric hepatectomy [10, 11, 12, 13, 14, 15].

With respect to the need for a standardized procedure for laparoscopic hemispheric hepatectomy, hemispheric hepatectomy is often performed by young surgeons, and an educational, standardized surgical procedure is needed. Indeed, many of the operations in the present study were performed by young training surgeons. In addition, determining the depth of dissection in laparoscopic hepatectomy is often difficult in segments 7 and 8, where the field of view is difficult to secure and the risk of positive resection margins is high. As described in detail in the Methods section, using our surgical technique, named the “can‐opener method”, the dorsal margin of the tumor is identified at the 3 o'clock liver dissection, which is the goal of liver dissection, allowing us to perform a resection that ensures a dorsal margin. In fact, all patients had negative resection margins in the present study.

Another benefit of standardizing surgical procedures is that it is helpful for facilitates surgical planning and allows for the sharing of complicated anatomical images with surgical staff. As mentioned in the Methods section, laparoscopic hemispheric hepatectomy requires cooperation between the surgeon and the assistant, especially in the direction of liver deployment by the assistant, which is essential for a smooth surgical procedure.

Relationships between the standardization of surgical procedures and improvements in surgical outcomes must be addressed. As mentioned in the Results section, we observed reductions in operating time, intraoperative blood loss volume, postoperative complications, and postoperative hospital stay after the surgical procedure was standardized. It is assumed that standardizing the procedure improved the learning curve of the surgical team, thereby improving surgical outcomes. In comparison with literature concerning anatomical resection for segment 7, the surgical outcomes of Group B, which accounted for the second half of the cases, showed a tendency toward less blood loss [9, 10].

The present study has several limitations. First, we assumed that our selection criteria for laparoscopic hemispheric hepatectomy in segments 7 and 8 did not influence our results because the same procedure was performed for locations other than segments 7 and 8, with no significant differences in short‐term results. Second, in terms of the indication criteria for laparoscopic hemispheric hepatectomy in segments 7 and 8 in our institution, in the case of surgery for tumors larger than 5 cm, the area of the liver to be resected is larger, and the surgical operation may be more complicated. In addition, the weight of the liver increases when the surgical field is developed on the resected side of the liver, including the tumor, making it difficult to develop a surgical procedure. In fact, for patients in our selection criteria, it was possible to perform a standardized laparoscopic hemispheric hepatectomy in 40 of 42 cases. Third, the present study was a retrospective single‐center study with a relatively small patient cohort. Therefore, these results require confirmation by additional multicenter large‐scale studies and prospective randomized controlled studies.

In conclusion, the short‐term results obtained using our own standardized technique, named the “can‐opener method”, have been satisfactory. Therefore, we found that our standardized surgical technique is an adequate surgical procedure for hemispheric hepatectomy in segments 7 and 8.

## Ethics Statement

Approval of the research protocol by an Institutional Review Board: The ethics committee of our institute approved this study.

## Conflicts of Interest

The authors declare no conflict of interest

## Consent

N/A.

## Clinical Trial Registration

N/A.
